# Suppression of Migration and Invasion by 4-Carbomethoxyl-10-Epigyrosanoldie E from the Cultured Soft Coral *Sinularia sandensis* through the MAPKs Pathway on Oral Cancer Cells

**DOI:** 10.1155/2024/6695837

**Published:** 2024-02-12

**Authors:** Rou-Yi Fang, Yueh-Wen Liu, Yih-Gang Goan, Jen-Jie Lin, Jui-Hsin Su, Wen-Tung Wu, Yu-Jen Wu

**Affiliations:** ^1^Department of Pharmacy, Kaohsiung Veterans General Hospital, Pingtung Branch, Kaohsiung, Taiwan; ^2^Department of Nursing, Meiho University, Pingtung, Taiwan; ^3^Department of Cosmetics and Fashion Styling, Cheng Shiu University, Kaohsiung, Taiwan; ^4^Division of Thoracic Surgery Department of Surgery, Pingtung Veterans General Hospital, Pingtung, Taiwan; ^5^Yu Jun Biotechnology Co., Ltd., Kaohsiung, Taiwan; ^6^National Museum of Marine Biology and Aquarium, Pingtung 94450, Taiwan; ^7^Department of Food Science and Nutrition, Meiho University, Pingtung 91202, Taiwan

## Abstract

The primary reason for cancer-related fatalities is metastasis. The compound 4-carbomethoxyl-10-epigyrosanoldie E, derived from the *Sinularia sandensis* soft coral species grown in cultures, exhibits properties that counteract inflammation. Moreover, it has been observed to trigger both apoptosis and autophagy within cancerous cells. This research focuses on examining the inhibitory impact of 4-carbomethoxyl-10-epigyrosanoldie E on the migration and invasion processes in Cal-27 and Ca9-22 oral cancer cell lines. To assess how this compound affects cell migration and invasion, the Boyden chamber assay was employed. Furthermore, Western blot analysis was utilized to explore the underlying molecular mechanisms. In a dose-dependent manner, 4-carbomethoxyl-10-epigyrosanoldie E notably decreased the levels of matrix metalloproteinase-2 (MMP-2) and MMP-9, along with urokinase-type plasminogen activator (uPA), in both Cal-27 and Ca9-22 cell lines. Conversely, it elevated the concentrations of tissue inhibitors of metalloproteinases-1 (TIMP-1) and TIMP-2. In addition, the treatment with this compound led to the inhibition of phosphorylation in extracellular signal-regulated kinase (ERK), p38, and c-Jun N-terminal kinase (JNK). It also curtailed the expression of several key proteins including focal adhesion kinase (FAK), protein kinase C (PKC), growth factor receptor-bound protein 2 (GRB2), Rac, Ras, Rho A, mitogen-activated protein kinase kinase kinase 3 (MEKK3), and mitogen-activated protein kinase kinase 7 (MKK7). Furthermore, the expression levels of IQ-domain GTPase-activating protein 1 (IQGAP1) and zonula occludens-1 (ZO-1) were significantly reduced by the compound. The ability of 4-carbomethoxyl-10-epigyrosanoldie E to inhibit the migration and invasion of Cal-27 and Ca9-22 oral cancer cells was observed to be dose dependent. This inhibitory effect is primarily attributed to the suppression of MMP-2 and MMP-9 expression, as well as the downregulation of the mitogen-activated protein kinase (MAPK) signaling pathway.

## 1. Introduction

Oral cancer most commonly develops from the squamous cells lining the mouth, with oral squamous cell carcinoma (OSCC) accounting for approximately 90% of these cases [1]. Major risk factors contributing to the development of oral cancer are the consumption of betel nuts, tobacco use, and alcohol intake. These factors are linked to a higher prevalence of the disease in males compared to the study by [2]. Early detection of oral cancer is often possible through routine physical exams, during which healthcare professionals look for early signs like white or red patches in the mouth's mucosa. The detection of any unusual lumps through palpation can also be an indicator of oral cancer [3].

Metastasis is a major factor leading to death in cancer patients, and its presence often signals how advanced the disease is and how well treatments are working. Gaining a deeper insight into the molecular processes that drive metastasis is crucial for creating effective medications for aggressive tumors [4]. Metastatic cancers, known for their widespread distribution and distinct genetic traits, frequently show resistance to conventional therapies. To address this challenge, treatments such as immunotherapy and chemotherapy are used systemically to curb the expansion of metastatic cancer cells [5].

Multiple pathways play key roles in controlling cell growth, differentiation, and cell death (apoptosis), and they are often involved in the spread of cancer (metastasis). One important pathway is the mitogen-activated protein kinase (MAPK) pathway, which includes the JNK, p38 MAPK, and ERK pathways. This pathway is critical in both the development and progression of cancer. Therefore, targeting the MAPK signaling pathway has become a major area of focus in cancer research and in the creation of new cancer treatments [6].

Soft corals from marine environments are notable for their richness in bioactive compounds. These compounds exhibit a range of biological activities including antimicrobial, antineoplastic, ichthyotoxic, cytotoxic, antiviral, and anti-inflammatory effects [7]. Among these, cembranoids, a category of monocyclic diterpenoids, are frequently encountered as secondary metabolites in both terrestrial and aquatic species. Their primary attribute is cytotoxicity [8, 9]. In a pivotal study by Lin et al. in 2014, the cembrane diterpenoid known as 11-epi-sinulariolide acetate, extracted from *Sinularia flexibilis*, a type of soft coral, was found to be effective in hampering cell migration and invasion in hepatic cancer models [10]. Following this, a 2017 study by Neoh et al. explored another cembrane diterpenoid, flaccidoxide-13-acetate, sourced from *Cladiella kashmani*. This compound exhibited inhibitory effects on the migration and invasion of human bladder cancer cells [11]. Subsequently, in 2018, a research led by Tsai et al. identified the cembrane-type diterpenoid 7-acetylsinumaximol B from *Sinularia sandensis*, demonstrating its apoptosis-inducing potential in gastric cancer cell lines [12].

In recent research, Chen et al. in 2020 identified that extracts from the cultured soft coral *Sinularia flexibilis*, specifically cembrane diterpenoids, exhibit cytotoxic properties against cancer cells and also possess properties counteracting acne [13]. Furthermore, a novel cembranoid, 4-carbomethoxyl-10-epigyrosanoldie E, derived from the cultured *Sinularia sandensis* species of soft coral, was found to have significant anti-inflammatory effects [14]. Expanding upon this, She et al. in 2022 revealed that this cembranoid not only induces apoptosis in oral cancer cells but also triggers autophagy. Their findings suggested that this dual effect is primarily due to mitochondrial dysfunction and endoplasmic reticulum stress, leading to cell cycle arrest and subsequent apoptosis [15].

In our current research, we delve into the influence and underlying mechanisms of 4-carbomethoxyl-10-epigyrosanoldie E on the migration and invasion processes in oral cancer cells. This investigation aims to contribute valuable insights to the field of antitumor drug development, potentially guiding the creation of novel therapeutic agents.

## 2. Materials and Methods

### 2.1. Chemicals and Reagents

The compound 4-carbomethoxyl-10-epigyrosanoldie E was extracted from the cultured soft coral species *Sinularia sandensis*, following a previously described method [15] and was dissolved in dimethyl sulfoxide (DMSO). Dulbecco's modified Eagle's medium, FBS (fetal bovine serum), penicillin, and streptomycin were sourced from Gibco BRL (Grand Island, NY, USA). The MTT reagent and other standard chemicals were acquired from Sigma-Aldrich Corporation (St Louis, MO, USA). Rabbit anti-human *β*-actin antibody and goat anti-rabbit IgG-conjugated horseradish peroxidase were procured from EMD Millipore (Billerica, MA, USA). For Western blot HRP detection, we used the chemiluminescent substrate from Pierce (Rockford, IL, USA). We obtained rabbit antibodies against human MEKK3, MKK7, GRB2, FAK, Ras, and RhoA from Epitomics Inc. (Burlingame, CA, USA); antibodies against human TIMP-1 and TIMP-2 from ProteinTech Group Inc. (Rosemont, IL, USA); and antibodies against human MMP-2, MMP-9, uPA, ERK and phosphorylated-ERK, P38 and phosphorylated-P38, JNK and phosphorylated-JNK, and c-Jun and phosphorylated-c-Jun from Cell Signaling Technology Inc. (Danvers, MA, USA).

### 2.2. Cell Culture

Oral squamous cell carcinoma cells, Cal-27 and Ca9-22, were obtained from the Taiwan Food Industry Research and Development Institute (Hsinchu, Taiwan). These cells were maintained at 37°C in a 5% CO_2_ environment, using Dulbecco's modified Eagle's medium (DMEM) supplemented with 10% FBS (v/v), 100 units/ml of penicillin, and 100 *μ*g/ml streptomycin. For the experiments involving the compound, cells were exposed to a DMSO vehicle as a control comparison.

### 2.3. Measurement of Cell Viability

Cell viability was evaluated using the MTT assay. To test the cytotoxic effects of drug treatments, cells seeded in 24-well plates were exposed to various concentrations of 4-carbomethoxyl-10-epigyrosanoldie E (2.5, 5, 7.5, and 10 *μ*M). Following a 24-hour incubation period, MTT solution (1 mg/ml) was added to each well and allowed to incubate for another 3 hours. Subsequently, the culture medium was removed, and the cells were solubilized in DMSO with a 10-minute shaking. Optical density (OD) values were then measured using a microplate reader (Bio-Rad, Hercules, CA, USA). This procedure was conducted in triplicate for each experiment.

### 2.4. Cell Migration and Invasion Assay

Cell migration was assessed using a technique as previously described in the method by [16]. We seeded 5 × 10^4^ cells, treated with the drug in serum-free media, into a Boyden chamber (Neuro Probe; Cabin John, MD, USA). These were then incubated at 37°C for 24 hours, allowing migrating cells to move through the membrane.

To evaluate cell invasion, we employed a method involving Transwell inserts with 8 *μ*m pore-size polycarbonate membrane filters, precoated with 0.5 mg/mL Matrigel, as previously described [16]. Cells suspended in serum-free media were placed in the upper chamber, while the lower chamber contained serum-enriched media. Following the incubation period, membranes from the bottom chamber were washed with PBS and treated with 1 mg/ml MTT solution and then incubated at 37°C for an hour. The resultant formazan crystals were dissolved in DMSO, and absorbance was measured at 550 nm.

### 2.5. Determination of MMP-2/-9 Activities by Gelatin Zymography

The activity of MMP-2/-9 enzymes was analyzed using gelatin zymography, as outlined in [17]. Cells were treated with varying concentrations of the drug for 24 hours, after which the culture media were gathered and concentrated using a speed vacuum. The concentrated samples were then run on a 10% native PAGE gel that included 0.2% gelatin. Postelectrophoresis, the gels underwent three wash cycles with a buffer containing 100 mM NaCl, 2.5% Triton X-100, and 50 mM Tris-HCl at pH 7.5. The gels were then incubated in a reaction buffer (200 mM NaCl, 1 mM CaCl_2_, 0.02% NaN_3_, 1 *μ*M ZnCl_2_, 2% Triton X-100, 50 mM Tris-HCl buffer, and pH 7.5) for 24 hours at 37°C. Postincubation, gels were stained using Coomassie blue R-250 and then destained. The MMP-2/-9 activity was quantified using Image J software (NIH, MD, USA).

### 2.6. Immunoblotting Analysis

Poststimulation, the cells were washed twice using ice-cold PBS and then lysed with 100 *μ*L of lysis buffer (containing 20 mM Tris/HCl pH 7.5, 125 mM NaCl, 1% Triton X-100, 1 mM MgCl_2_, 25 mM *β*-glycerophosphate, 50 mM NaF, 100 *μ*M Na_3_VO_4_, 1 mM PMSF, 10 *μ*g/mL leupeptin, and 10 *μ*g/mL aprotinin). The proteins were denatured, run on a 10% SDS-PAGE, and then transferred to a PVDF membrane. The membrane was blocked for nonspecific interactions using TBST (50 mM Tris-HCl, pH 7.5, 150 mM NaCl, and 0.1% Tween 20) with 5% nonfat milk for 1 hour at room temperature. Following the primary antibody incubation, the membranes underwent three TBST washes and were incubated with the secondary antibody for 1 hour. After another set of three washes in TBST, protein bands were visualized using ECL® reagent.

### 2.7. Statistical Evaluation

In this research, measurement values are presented as the average ± standard error of the mean (S.E.M.) from a minimum of three independent experiments, each conducted in duplicate. To determine the statistical relevance of the differences observed, analysis of variance (ANOVA) was utilized. A *P* value of less than 0.05 was deemed to be statistically significant.

## 3. Results

### 3.1. Inhibitory Effects of 4-Carbomethoxyl-10-Epigyrosanoldie E on Cell Migration and Invasion

**Figure 1:**
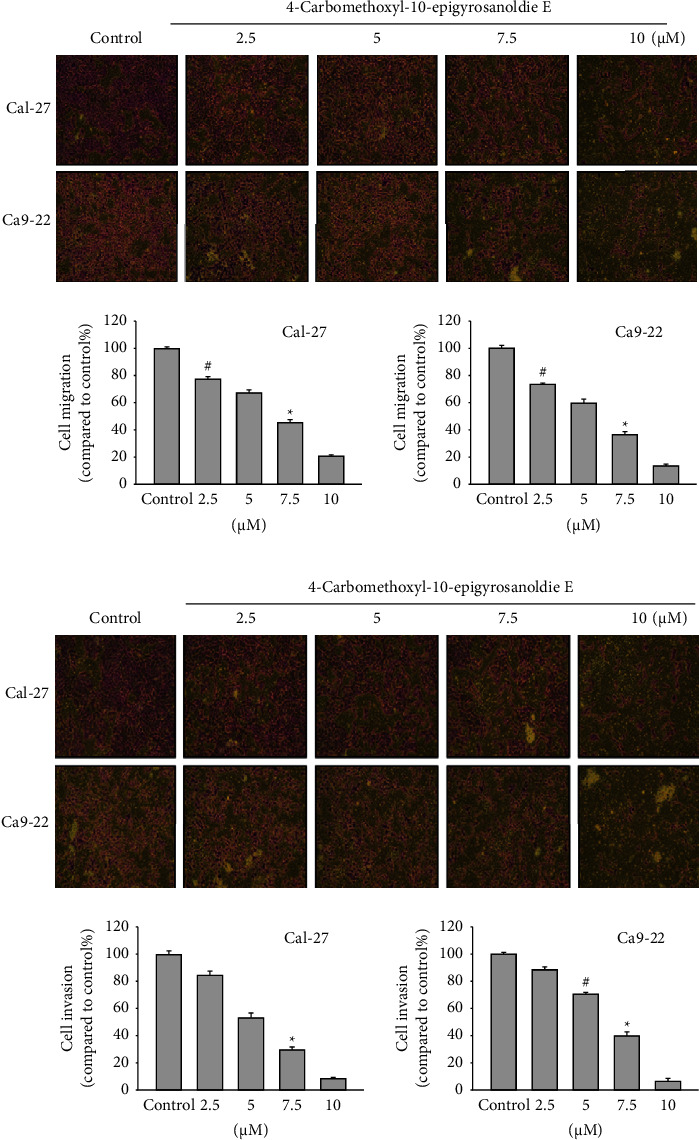
4-Carbomethoxyl-10-epigyrosanoldie E inhibits Cal-27 and Ca9-22 cell migration (a) and cell invasion (b). Control means cells were treated with DMSO vehicle control (*n* = 3; three independent experiments) (^#^*p* < 0.05 and ^*∗*^*p* < 0.001).

Tests examining cell toxicity and structure revealed that the compound 4-carbomethoxyl-10-epigyrosanoldie E effectively reduced cell growth in both Ca9-22 and Cal-27 cell lines [15]. To prevent low cell viability due to high concentrations of 4-carbomethoxyl-10-epigyrosanoldie E, we used doses ranging from 0 to 10 *μ*M in our experiments. We assessed the impact of 4-carbomethoxyl-10-epigyrosanoldie E on the movement and invasion abilities of Cal-27 and Ca9-22 oral cancer cells using a Boyden chamber. For cell migration, we tested the compound at various concentrations (2.5, 5, 7.5, and 10 *μ*M) and observed an increase in its inhibitory effect with higher concentrations. This inhibition followed a dose-dependent pattern, where cell migration activity was reduced to only 20% and 15% at a concentration of 10 *μ*M in Cal-27 and Ca9-22 cells, respectively (Figure 1(a)). Regarding cell invasion, we used the same range of concentrations for the tests. The findings indicated that as the concentration of 4-carbomethoxyl-10-epigyrosanoldie E increased, there was a notable reduction in the invasion capabilities of both Cal-27 and Ca9-22 cells. This demonstrated a pattern where the extent of inhibition was dependent on the concentration of the compound (Figure 1(b)). At a concentration of 10 *μ*M, the compound reduced the invasion activity to just 10% in Cal-27 cells and 8% in Ca9-22 cells, suggesting its effectiveness in hindering the invasion of oral cancer cells. These results indicate that 4-carbomethoxyl-10-epigyrosanoldie E potentially impacts both the migration and invasion abilities of Cal-27 and Ca9-22 oral cancer cells.

### 3.2. Effects of 4-Carbomethoxyl-10-Epigyrosanoldie E on the Expression Levels of MMP-2, MMP-9, uPAR, TIMP-1, and TIMP-2

MMP-2 and MMP-9 are part of the matrix metalloproteinase (MMP) family, known for their ability to break down polymeric collagen and the extracellular matrix (ECM), factors linked to cancer metastasis and angiogenesis. The activities of MMP-2 and MMP-9 were assessed through gelatin zymography. The analysis revealed a decline in the activities of MMP-2 and MMP-9 with increasing concentrations of 4-carbomethoxyl-10-epigyrosanoldie E (2.5, 5, 7.5, and 10 *μ*M) (data not shown), suggesting that the compound effectively inhibits these enzymes. Subsequently, we explored the impact of 4-carbomethoxyl-10-epigyrosanoldie E on the expression of MMP-2, MMP-9, and other related proteins in cell migration and invasion, utilizing Western blot analysis for this purpose.

**Figure 2:**
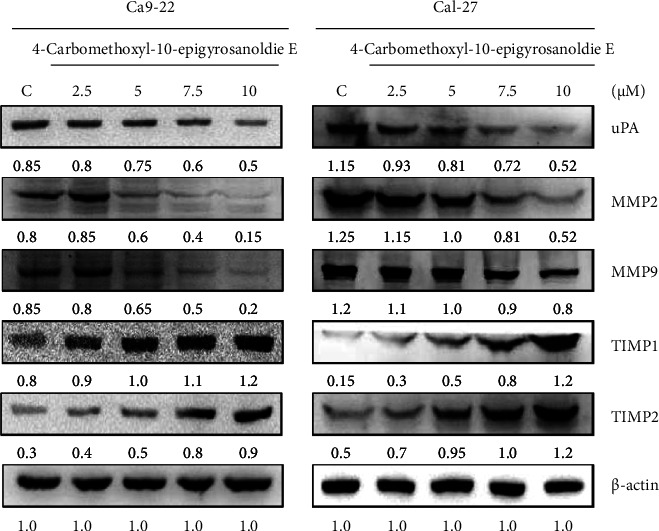
4-Carbomethoxyl-10-epigyrosanoldie E suppressed the expressions of MMP-2, MMP-9, and uPAR protein and augmented the expressions of TIMP-1 and TIMP-2 protein. The expression levels of MMP-2, MMP-9, uPAR, TIMP-1, and TIMP-2 were analyzed by Western blotting. The total cell lysates of Cal-27 and Ca9-22 cells treated with 4-carbomethoxyl-10-epigyrosanoldie E, respectively, were analyzed. C means cells were treated with DMSO vehicle only. *β*-Actin was used as the protein loading control.

Figure 2 illustrates that in both Cal-27 and Ca9-22 cells, the levels of MMP-2, MMP-9, and uPA were reduced as the concentration of 4-carbomethoxyl-10-epigyrosanoldie E increased. In contrast, the levels of TIMP-1 and TIMP-2 showed an increase.

### 3.3. Effects of 4-Carbomethoxyl-10-Epigyrosanoldie E on the MAPK Signaling Pathway

**Figure 3:**
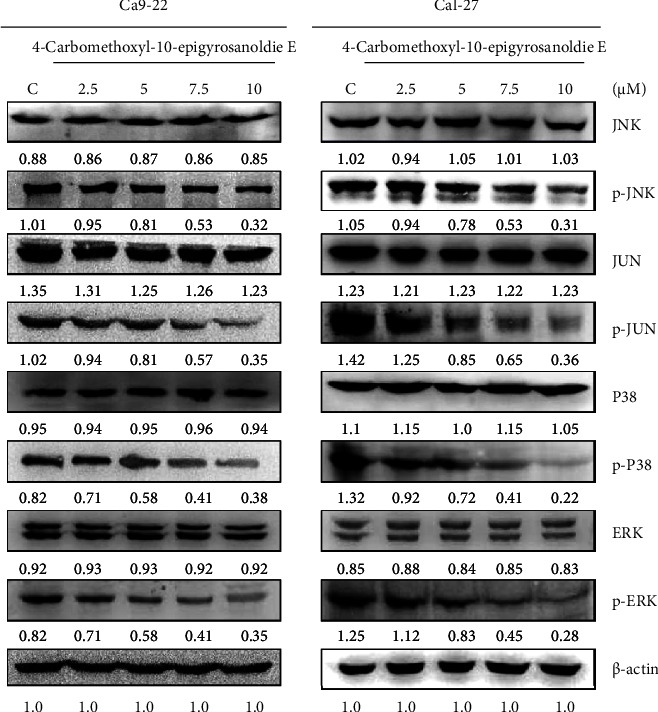
4-Carbomethoxyl-10-epigyrosanoldie E regulated the expression levels of the MAPK signaling pathway. Western blotting analysis demonstrated the expression levels of p-JNK, p-c-Jun, p-P38, and p-ERK in Cal-27 and Ca9-22 cells treated with 4-carbomethoxyl-10-epigyrosanoldie E, respectively. C means cells were treated with DMSO vehicle only. *β*-Actin was used as the protein loading control.

The mitogen-activated protein kinase (MAPK) pathway plays a vital role in cellular processes such as growth, differentiation, and development. Dysregulation of this pathway can lead to improper cell differentiation and apoptosis, potentially causing cells to transform malignantly and proliferate uncontrollably. An altered MAPK pathway could thus enhance the metastatic and invasive capabilities of cancer cells. Western blot analysis revealed that in Cal-27 and Ca9-22 cells treated with 4-carbomethoxyl-10-epigyrosanoldie E, the overall MAPK protein levels remained relatively unchanged. However, the phosphorylation of JNK, c-Jun, P38, and ERK decreased as the concentration of 4-carbomethoxyl-10-epigyrosanoldie E increased (Figure 3). Since activated MAPK proteins are known to promote cancer cell proliferation, antiapoptosis, and tumor spread, these findings suggest that 4-carbomethoxyl-10-epigyrosanoldie E could influence cancer cell migration and invasiveness through the MAPK pathway.

### 3.4. Effects of 4-Carbomethoxyl-10-Epigyrosanoldie E on the Expression of Cell Migration- and Invasion-Associated Proteins

We further investigated how 4-carbomethoxyl-10-epigyrosanoldie E impacts the levels of proteins such as FAK, PKC, GRB2, Rac, Ras, RhoA, MEKK3, and MKK7, which are upstream regulators in the MAPK pathway and play a role in controlling the expression of MMPs involved in cancer cell metastasis. Western blot analysis revealed a decrease in the expression of these upstream MAPK molecules with higher doses of 4-carbomethoxyl-10-epigyrosanoldie E, indicating their potential link to reduced cell migration and invasion.

**Figure 4:**
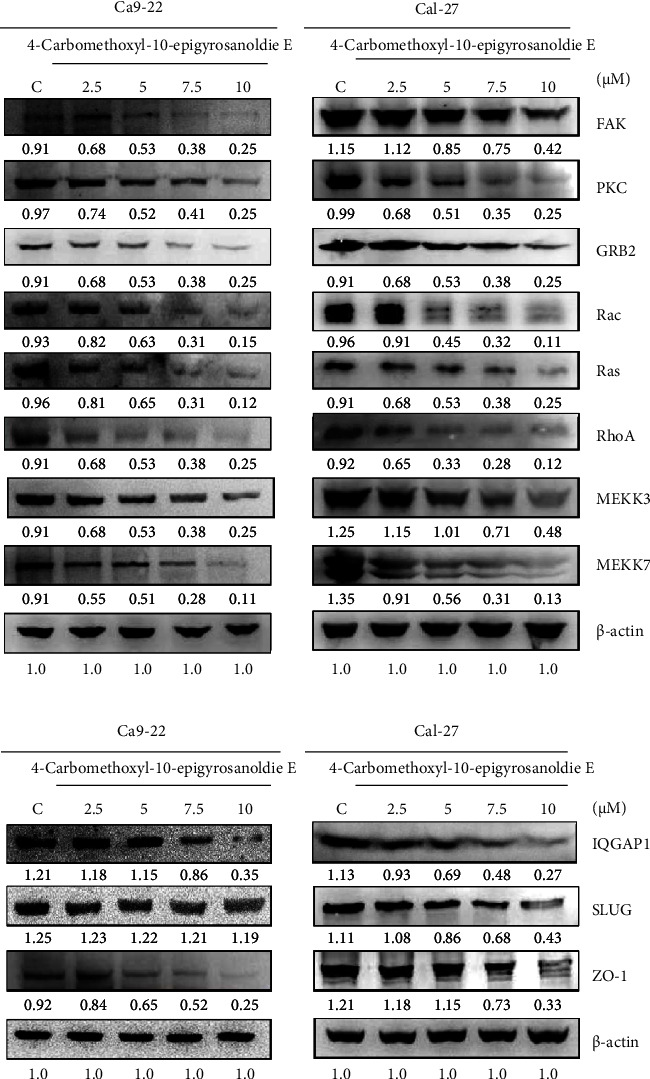
4-Carbomethoxyl-10-epigyrosanoldie E reduced the expression levels of proteins associated with cell migration and invasion. (a) Western blotting analysis examined the expression levels of FAK, PKC, GRB2, Rac, Ras, RhoA, MEKK3, and MKK7 in Cal-27 and Ca9-22 cells treated with 4-carbomethoxyl-10-epigyrosanoldie E (2.5–10 *μ*M), respectively. (b) Expression levels of IQGAP1, SLUG, and ZO-1 in Cal-27 and Ca9-22 cells treated with 4-carbomethoxyl-10-epigyrosanoldie E (2.5–10 *μ*M), respectively. C means cells were treated with DMSO vehicle only. *β*-Actin was used as the protein loading control.

Moreover, the study also showed that at elevated concentrations of the compound, the levels of other metastasis-related proteins, such as IQGAP1 and ZO-1, were significantly diminished. However, the expression of SLUG remained unchanged, as illustrated in Figure 4(b).

## 4. Discussion

Cancer's ability to migrate to different body parts, termed metastasis, is a primary reason for cancer-related deaths. This process allows cancer cells to enter the bloodstream, reach distant organs, and establish new malignant growths. Understanding the intricate process of metastasis is crucial for effectively managing the spread of cancer cells. Our research indicates that the compound 4-carbomethoxyl-10-epigyrosanoldie E demonstrates inhibitory effects on the migration and invasion of Cal-27 and Ca9-22 cells, particularly at concentrations above 5 *μ*M.

MMPs, which are proteases containing zinc ions, play a key role in degrading components of the extracellular matrix, including collagen, laminin, and proteoglycans. This process is vital in facilitating the metastasis of cancer cells [18]. Earlier studies have shown that extracts from *Dioscorea nipponica* Makino (DNE) suppress the activity of the matrix metalloproteinase-2 (MMP-2) enzyme, as well as its RNA and protein levels, in oral cancer cell lines Ca9-22 and Cal-27 [19]. In studies using mice lacking MMP-9, there was a notable reduction in metastatic colony formation from melanoma and carcinoma cells [20]. High levels of MMP-2, MMP-9, and vascular endothelial growth factor (VEGF) have been closely linked to cell growth, dissemination, metastasis, and the development of new blood vessels in gastric cancer [21]. The interaction between matrix metalloproteinases (such as MMP-2 and MMP-9) and VEGF is a key focus in cancer research, given their combined impact on cancer progression and metastasis. VEGF, a vital angiogenesis-promoting signaling protein, is overexpressed in many cancers, aiding in tumor growth and metastasis by fostering new blood vessel formation.

Research has demonstrated that dihydromyricetin impedes cell migration and MMP-2 expression in human nasopharyngeal carcinoma, with this action mediated through the ERK1/2 signaling pathway [22]. In addition, there is an interactive relationship among EGF, growth factor, metallothionein 2A, and MMPs in malignant tumors. Dias and colleagues found that reducing metallothionein 2A levels using siRNA lowered MMP-9 and EGF protein levels, leading to decreased proliferation, migration, and invasion in human oral squamous cell carcinoma cells [23]. Tissue inhibitors of metalloproteinases (TIMPs) regulate MMPs, and changes in TIMP levels are crucial as they directly affect MMP activity. Monitoring changes in the MMP/TIMP profile could help predict the effectiveness of cancer treatments [24].

Neoh et al. found that an extract from soft coral, flaccidoxide-13-acetate, curbed migration and invasion in T24 and RT4 human bladder cancer cells. They observed an increase in TIMP-1 and TIMP-2 protein levels alongside heightened flaccidoxide-13-acetate concentrations, while MMP-2 and MMP-9 expressions declined simultaneously [11]. In addition, the urokinase-type plasminogen activator (uPA) receptor (uPAR), known for its high expression in aggressive tumors, showed a drop in levels, signifying treatment efficacy [25]. In our study, increasing amounts of 4-carbomethoxyl-10-epigyrosanoldie E led to a 5.3-fold decrease in MMP-2 and a 4.25-fold decrease in MMP-9, while TIMP-1 and TIMP-2 expressions rose 8-fold and 3-fold, respectively, as shown in Figure 2. These findings imply that 4-carbomethoxyl-10-epigyrosanoldie E can hinder the migration and invasion of Cal-27 and Ca9-22 oral cancer cells. Given the crucial role of MMP-2 and MMP-9 in tumor migration and invasion, research has substantiated that inhibiting the MAPK (JNK/P38/ERK) pathway and reducing MMP-2, MMP-9, and uPA protein expression can effectively prevent cancer cell migration and invasion [26–28].

The influence of 4-carbomethoxyl-10-epigyrosanoldie E on cell metastasis and invasion, potentially via the MAPK signaling pathway, was investigated in Cal-27 and Ca9-22 oral cancer cells. Western blot analysis revealed that as the concentration of 4-carbomethoxyl-10-epigyrosanoldie E increased, there was a reduction in the expression of phosphorylated forms of JNK, c-Jun, P38, and ERK (Figure 3). This suggests that the compound may impede cancer cell migration and invasion by targeting the MAPK pathway. Similarly, sinulariolide, extracted from *Sinularia flexibilis*, has been shown to suppress MMP-2 and MMP-9 expression via the MAPK pathway in liver and gastric cancer cells, thereby diminishing cancer cell migration and invasion [29, 30]. Marine soft corals, including compounds such as 11-epi-sinulariolide acetate [10], flaccidoxide-13-acetate [11], 7-acetylsinumaximol B [12], and 4-carbomethoxyl-10-epigyrosanoldie E, are known for their rich bioactive secondary metabolites, particularly cembrane diterpenoids with cytotoxic properties. In addition, triterpenes from *Ganoderma lucidum* have been found to trigger apoptosis in lung cancer and other tumors [31].

FAK plays critical roles in cell adhesion and migration. Research by Chan et al. indicated that reducing FAK levels may lead to lower invasion rates in breast cancer cells [32]. Multiple studies have demonstrated that FAK inhibitors can lessen tumor growth and metastasis, with FAK known to regulate MMP expression in tumor cells and promote their survival, migration, proliferation, and angiogenesis [33]. Sieg et al. found that reintroducing FAK into FAK-deficient cells restored their migration capabilities by integrating growth factor and integrin signals [34]. Our study revealed that 4-carbomethoxyl-10-epigyrosanoldie E suppresses FAK protein expression in oral cancer cells, impacting downstream MAPK proteins and MMPs (Figure 4). In addition, the compound reduced the expression of proteins involved in cancer cell migration and invasion, such as PKC, GRB2, Rac, Rho A, Ras, MKK7, and MEKK3. FAK has been suggested to activate PKC, with its overexpression enhancing cell proliferation through the PKC pathway [35]. Schlaepfer and Hunter's studies showed that FAK phosphorylation enables its binding to Grb2, which then activates the downstream Ras signal [36]. Grb2 is crucial for linking cell surface receptors to downstream proteins, playing a key role in cell cycle progression and actin-based cell motility [37]. Cell migration involves the cooperative action of GTP-binding proteins Rho and Rac, with Rho promoting actin polymerization and contraction and Rac managing pseudopod and membrane ruffle formation, thereby orchestrating the dynamic interaction between actin and cell adhesion [38]. Our findings confirm that 4-carbomethoxyl-10-epigyrosanoldie E inhibited both Rho and Rac, reducing oral cancer cell migration and invasion.

Metastasis in oral cancer, like in many other types of cancer, is a complex and multifactorial process involving various signaling pathways and mechanisms. While the MAPK (mitogen-activated protein kinase) signaling pathway is one of the pathways associated with cancer metastasis, there are several other pathways and factors involved. For example, curcumin was reported to shown anti-inflammatory, antioxidant, and antitumor activities with therapeutic benefits against various cancers. It can regulate multiple signaling pathways, such as PI3K/Akt, Wnt/beta-catenin, JAK/STAT, p53, MAPKs, and NF-kB, which are involved in cancer cell proliferation, metastasis, apoptosis, angiogenesis, and autophagy [39]. Another potential pathway is the Notch signaling pathway which is an evolutionarily ancient mechanism which intricated in cell-cell communication and it plays a crucial role in various developments in malignancies. Inactivating mutations of Notch targets are present in about 10% of the cases of squamous cell carcinoma of the skin, oral cavity, and esophagus, rendering it one of the most frequently mutated genes in oral squamous cell carcinoma [40]. Curcumin has also been shown to inhibit migration and invasion in rodent retina ganglion N18 cells by suppressing RhoA, MMP-2, and MMP-9 expression [41]. RhoA is known to trigger MMP-9 expression in human microvascular endothelial cells, promoting cell migration and invasion [42]. Thus, when 4-carbomethoxyl-10-epigyrosanoldie E inhibits RhoA expression, this leads to a decrease in MMP-2 and MMP-9 levels, impacting the migration and invasion of Cal-27 and Ca9-22 oral cancer cells. Thant et al. noted that Ras regulates MMP expression [43]. Our findings also indicate that 4-carbomethoxyl-10-epigyrosanoldie E treatment inhibits Ras expression in oral cancer cells, affecting downstream MMP proteins and reducing cancer cell migration and invasion.

The migration of cancer cells is influenced by the IQGAP1 protein. Research by Chellini et al. indicated that the silencing of IQGAP1 led to reduced cell mobility due to its disrupted interaction with beta-catenin [44]. SLUG, a zinc finger transcription factor, functions as a transcriptional repressor. Hajra et al. demonstrated that SLUG's binding to E-box elements in the E-cadherin promoter suppressed E-cadherin transcription, impacting breast cancer cell growth [45]. ZO-1, crucial in regulating tight junctions, plays a role in cell transformation and migration [46]. Our study found that 4-carbomethoxyl-10-epigyrosanoldie E significantly reduced the expression of IQGAP1 and ZO-1 (as shown in Figure 4(b)), which in turn suppressed cell migration. However, SLUG expression remained unchanged, suggesting a reduced proliferation ability. These findings elucidate how 4-carbomethoxyl-10-epigyrosanoldie E inhibits cell migration and invasion in oral cancer cells.

## 5. Conclusion

**Figure 5:**
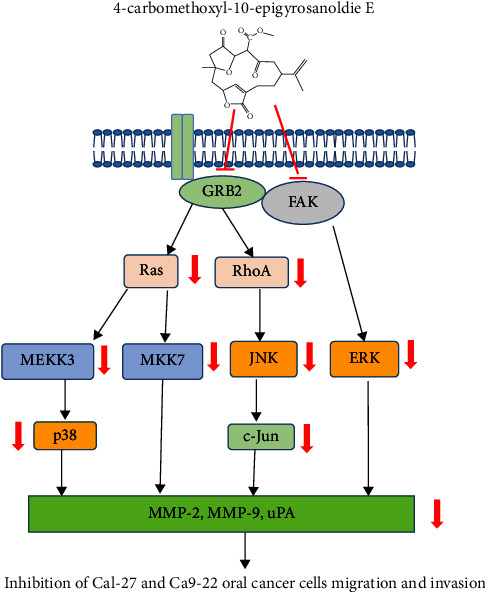
The mechanism of 4-carbomethoxyl-10-epigyrosanoldie E inhibition of Cal-27 and Ca9-22 oral cancer cell migration and invasion mediated by the MAPK pathway.

The compound 4-carbomethoxyl-10-epigyrosanoldie E, derived from the cultured soft coral *Sinularia sandensis*, effectively reduced migration and invasion of Cal-27 and Ca9-22 oral cancer cells, showing concentration-dependent effects. As depicted in Figure 5, its regulatory action primarily involves targeting upstream proteins such as FAK, Grb2, Ras, and RhoA, leading to the inhibition of the MAPK pathway. Consequently, this results in decreased levels of MMP-2, MMP-9, and uPAR proteins, curtailing the migratory and invasive capabilities of these oral cancer cells. While in vitro cell line studies are fundamental for preliminary research, they might not completely mirror the complexity found in vivo. Our findings indicate the potential of 4-carbomethoxyl-10-epigyrosanoldie E as an oral cancer metastasis treatment, though caution is advised in extrapolating these results to whole organisms. Further research, including animal studies or clinical trials, is necessary to confirm and expand upon our in vitro observations.

## Data Availability

The data generated used to support the findings of this study are included within the article.
